# A Search Advantage for Horizontal Targets in Dynamic Displays

**DOI:** 10.1177/20416695211004616

**Published:** 2021-04-13

**Authors:** Ian M. Thornton, Quoc C. Vuong, Karin S. Pilz

**Affiliations:** Department of Cognitive Science, Faculty of Media and Knowledge Sciences, University of Malta, Msida, Malta; Biosciences Institute, School of Psychology, 5994 Newcastle University, UK; Department of Experimental Psychology, 3647 University of Groningen, the Netherlands

**Keywords:** horizontal advantage, visual search, attention, orientation, anisotropy

## Abstract

Several lines of evidence point to the existence of a visual processing advantage for horizontal over vertical orientations. We investigated whether such a horizontal advantage exists in the context of top-down visual search. Inspired by change detection studies, we created displays where a dynamic target -- a horizontal or a vertical group of five dots that changed contrast synchronously -- was embedded within a randomly flickering grid of dots. The display size (total dots) varied across trials, and the orientation of the target was constant within interleaved blocks. As expected, search was slow and inefficient. Importantly, participants were almost a second faster finding horizontal compared to vertical targets. They were also more efficient and more accurate during horizontal search. Such findings establish that the attentional templates thought to guide search for known targets can exhibit strong orientation anisotropies. We discuss possible underlying mechanisms and how these might be explored in future studies.

When observers search for a known target, their behaviour is thought to be guided in a top-down manner by attentional templates held in working memory (Desimone & Duncan, 1995; Duncan & Humphreys, 1989; Grubert & Eimer, 2020; Wolfe & Horowitz, 2017). In this study, we asked whether orientation anisotropies known to affect other aspects of visual processing (e.g., Annis & Frost, 1973; Appelle, 1972; Barnas & Greenberg, 2016; Carrasco et al., 2004; Coppola & White, 2004; Maloney & Clifford, 2015; Pilz et al., 2017) also extend to attentional templates. Specifically, we wanted to know whether guided search for a horizontal target is faster and more efficient than search for a vertical target.

A range of evidence supports a general anisotropy favouring “horizontal” over “vertical” in the visual system. To begin with, an increased area of cortical volume is allocated to horizontal and near horizontal orientations (Coppola & White, 2004). In humans, horizontally oriented gratings also elicit differential neuroimaging responses compared to vertical and oblique ones (Maloney & Clifford, 2015; Mannion et al., 2010). Behaviourally, eye movements (Amor et al., 2016; Ke et al., 2013; Rottach et al., 1996), motion discrimination (Pilz et al., 2017; Pilz & Papadaki, 2019), and attention cueing (e.g., Barnas & Greenberg, 2016; Chen & Cave, 2019; Clevenger et al., 2019; Eppinger et al., 2011; Greenberg et al., 2014; Pilz et al., 2012; Tse et al., 2003) all exhibit a horizontal advantage. Explanations for such orientation preference typically revolve around the idea that horizontal objects/information may be more prevalent or more relevant in our visual world (e.g., Pilz et al., 2020; Rottach et al., 1996).

Despite the above evidence for a general horizontal advantage, to our knowledge, the question of whether top-down search also shows such an effect has not been previously addressed. Inspired by the search-for-change literature (Rensink, 2000, 2002), we created displays in which low-level cues to target location were rendered ineffective. Within dense grids of contrast modulating dots, five contiguous target elements were constrained to change synchronously (Figure 1; https://maltacogsci.org/flickersearch/). In different blocks of trials, the target elements could either be horizontally or vertically oriented.

**Figure 1.**
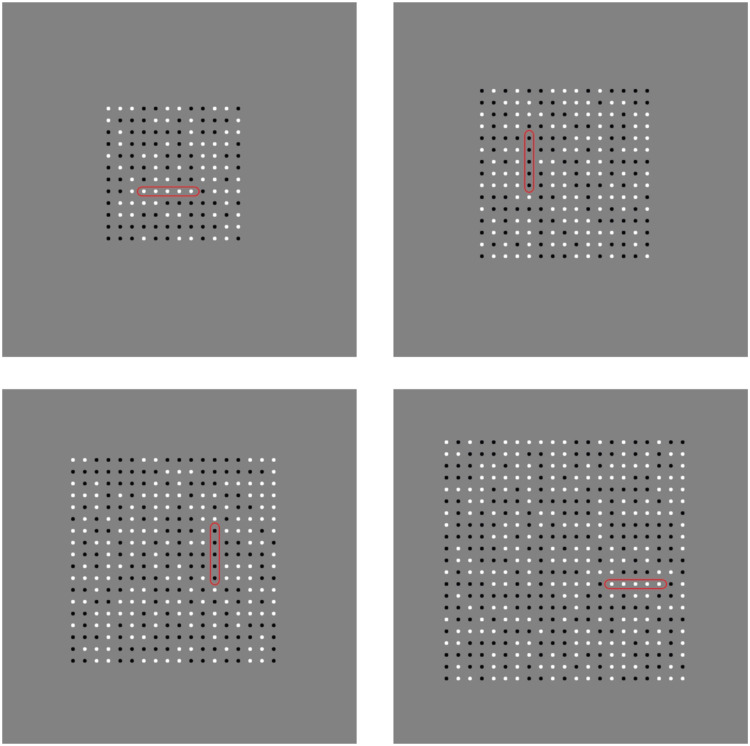
Static example snapshots of the search display. Each panel represents a single trial, which could contain 12 × 12, 15 × 15, 18 × 18, or 21 × 21 elements. Elements flickered randomly between black and white at a rate of 10 Hz. Targets elements (here highlighted in red) flickered synchronously with a pattern that was determined randomly on a trial by trial basis. For dynamic versions of these displays, see https://maltacogsci.org/flickersearch

The dynamic nature of both the target elements and the distracting dot fields ensured that bottom-up, feature-based cues to orientation that might lead to “pop-out” were rendered ineffective (Treisman & Souther, 1985). Specifically, due to the random nature of the flickering display, at any moment in time, multiple contiguous five dot sequences of each orientation were always present (see Figure 1), with the “singleton” nature of the true target only evolving over time. In addition, the multiple transients associated with the flickering of individual dots would effectively mask the location of the synchronously changing target (O’Regan et al., 1999). We also made sure that target location within the visual field was unpredictable and independent from the horizontal and vertical meridians, removing any potential interactions between target orientation and spatial layout (Carrasco et al., 2004; Hughes & Zimba, 1987; Kristjánsson & Sigurdardottir, 2008; Mackeben, 1999; Previc & Naegele, 2001).

We measured response time (RT), search efficiency, and accuracy while participants searched for dynamic orientation targets across changes in display size. Each participant completed two blocks of each target orientation, with block type interleaved (i.e., HVHV or VHVH). Our main question was whether a horizontal advantage would be found with this form of top-down, effortful search. By repeating target blocks, we hoped to shed light on the underlying nature of any observed advantage. That is, if an advantage can be eliminated with practice, then this would argue against a purely physiological locus for the effect.

## Methods

### Participants

A total of 15 participants (mean age = 21.2 years, *SD* = 2.5; 6 females and 9 right handed) were recruited from the University of Malta academic community. Group sample size was determined prior to data collection based on the effect size for the difference in horizontal versus vertical search slopes observed during a pilot study. In this pilot study (*N* = 6, see Supplementary Material), the observed effect size was 0.99 (Cohen’s *d*; mean difference divided by the pooled standard deviation). We used the G*Power 3.1 software package (Faul et al., 2009) to conduct a priori power analysis based on this estimate, with a power of 0.95 and an alpha of .05. This analysis suggested a minimum sample size of 13 participants. As this was our first main data collection exercise with this paradigm, we decided to be conservative and include an additional two participants beyond the suggested minimum in order to anticipate any potential technical or measurement issues with the task. All participants had self-reported normal or corrected-to-normal vision and gave written informed consent before taking part in the study. They were naïve as to the purpose of the research and were not experienced psychophysical observers. Participants received €8 compensation for taking part in the study. All methods and procedures conformed to the Ethics and Data Protection Guidelines of the University of Malta.

### Equipment

Participants were tested individually in a dimly lit experimental booth. The experiment was conducted using a 15 in. MacBook Pro (OS X 10.10), connected to a BENQ LCD monitor. The monitor had a visible viewing area of 53 × 29 cm and was configured with a resolution of 1,600 × 900 pixels and a simulated refresh rate of 60 Hz. Responses were made with a standard USB keyboard and mouse separate from the laptop. Participants sat approximately 60 cm from the monitor. Custom code was written in MATLAB, using the Psychophysics Toolbox (version 3; Brainard, 1997; Kleiner et al., 2007; Pelli, 1997). Copies of the experimental scripts are available on request.

### Stimuli

The stimuli consisted of square grids of dots that randomly changed polarity between black and white. Figure 1 illustrates the static layout of these displays, while the online videos (https://maltacogsci.org/flickersearch/) provide a clearer impression of their dynamic nature. The square grids could have 12, 15, 18, or 21 elements for the rows and columns, leading to 144, 225, 324, or 441 total elements, respectively. The overall grid height/width was either 4, 5, 6, or 7 cm, which subtended 3.8°, 4.8°, 5.7°, or 6.7° visual angle, respectively. The grids were centred on the screen which had a uniform middle-grey background. Each element in the grid was rendered as a disc with a diameter of 5 pixels (0.02° visual angle). The vertical and horizontal spacing between elements was 20 pixels (0.95° visual angle).

The initial polarity of each background element (black or white) was randomly determined on each trial. Each of these nontarget elements had an equal probability of switching or maintaining polarity independently of the other elements. This polarity-change decision occurred with a temporal frequency of 10 Hz.

Within the grid on each trial, there was a target that consisted of five consecutive elements (1.9°/2 cm). The initial contrast polarity of each element in the target was identical and subsequent polarity changes were synchronised. The rate of polarity change within the target was again 10 Hz, but the pattern of change was randomly predetermined at the start of each trial and was completely independent of the other background elements. The pattern of polarity changes within the target repeated every 2 seconds, providing a stable, spatiotemporal signature of change that could be used to guide search within, but not across, trials.

Across alternating blocks (counterbalanced), the target dots were either organised horizontally, within a row, or vertically, within a column of the grid. The position of the target within the grid was randomly determined on a trial by trial basis.

### Task

Participants were informed about the target orientation prior to the start of each block. Their task was to locate the position of the target within the grid as quickly and accurately as possible. They were instructed to press the spacebar as soon as they detected the target. This key press stopped the animation on the presented frame, which effectively rendered the target invisible. The RT on each trial was measured from the stimulus onset to the spacebar press.

Following the key press, a 10-pixel red dot (0.04° visual angle) was presented at the centre of the screen, superimposed on the grid. Participants used the mouse to move the red dot and click anywhere within the five elements comprising the target. As long as the clicked location was closer to a target element than to any other item in the grid, the selection was deemed to be correct. If no key press was made within 20 seconds, the next trial began automatically. There was a 500-ms intertrial interval. Responses were considered errors if participants clicked on or near nontarget elements or did not respond within 20 seconds. If they made an error, a short auditory feedback tone (150 ms; 400 Hz) was played.

### Design

Participants completed four blocks of trials, each containing 160 trials. The target orientation (horizontal or vertical) was fixed on a given block. The orientation on the first block was counterbalanced across participants and then alternated between each block. Within a block of trials, the 40 repetitions of each of the four display sizes (12, 15, 18, and 21 elements) were randomised for each participant.

### Data Analysis

Data from one participant was excluded as their performance in Block 1 did not exceed 50% correct, suggesting they were not able to follow instructions. We did not replace the participant, and analysis was conducted on the data from the remaining 14 participants. We calculated the median RT from correct trials and percentage correct (i.e., accuracy) as a function of target orientation, display size, and block. To assess search efficiency, we computed search slopes by regressing the median correct RT to display size. These slopes were analysed using a 2 (Orientation: horizontal or vertical) × 2 (Block: first or second block for each orientation) repeated measures analysis of variance (ANOVA). To characterise the absolute speed of responses, we also submitted median correct RTs to a 2 (Orientation) × 2 (Block) × 4 (Display size) repeated measures ANOVA. Although we expected accuracy to be close to ceiling levels, we used the same 2 (Orientation) × 2 (Block) × 4 (Display size) ANOVA to test for possible speed-accuracy trade-offs. Greenhouse–Geisser correction was used for any violations of sphericity. An alpha of .05 was used as our level of significance, and Bonferroni correction was used for post hoc comparisons.

### Data Availability Statement

The experimental scripts, raw data, and analysis tables have been uploaded to the OSF page associated with this article, which can be accessed at https://osf.io/vwtmk/

## Results

**Figure 2.**
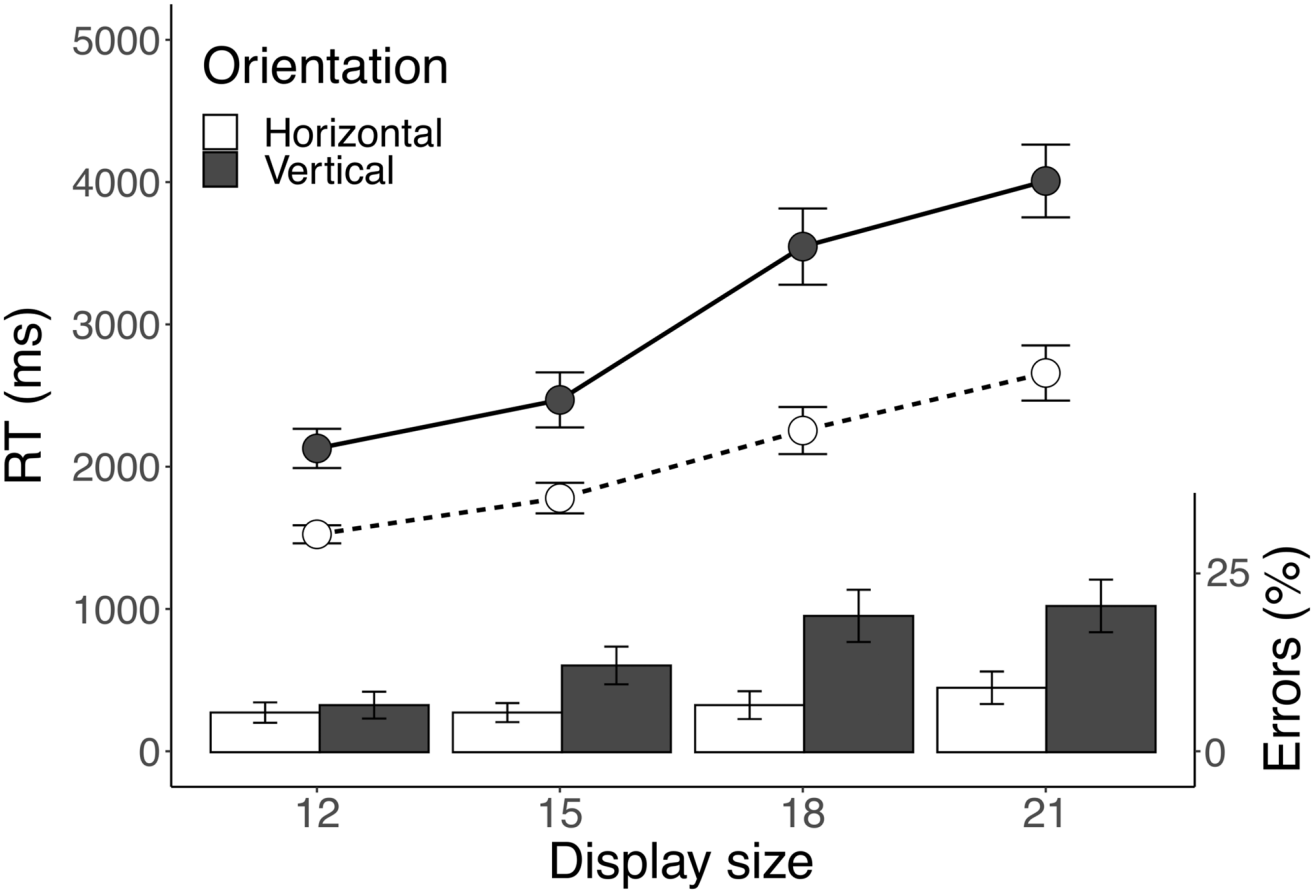
Median RT and error rates as a function of orientation and display size. Error bars represent standard error of the mean. RT = response time.

Figure 2 shows median correct RT and error rates as a function of orientation and display size. As expected, search was slow and inefficient, with the fastest responses remaining well above 1 second, increasing to over 4 seconds. Responses to horizontal targets (2.1 s) were faster than responses to vertical targets (3.0 s), *F*(1, 13) = 28.51, *p* < .001, $\eta_{p}^{2}$ = 0.69. There was no speed-accuracy trade-off, as accuracy showed the same horizontal advantage, *F*(1, 13) = 5.96, *p* = .03, $\eta_{p}^{2}$ = 0.31 (see Supplementary Materials for full analysis tables). Search slopes for horizontal targets (0.4 s/display size) were also significantly shallower than for vertical targets (0.6 s/display size), indicating more efficient localisation of horizontal targets, *F*(1,13) = 13.91, *p* = .003, $\eta_{p}^{2}$ = 0.52.

**Figure 3.**
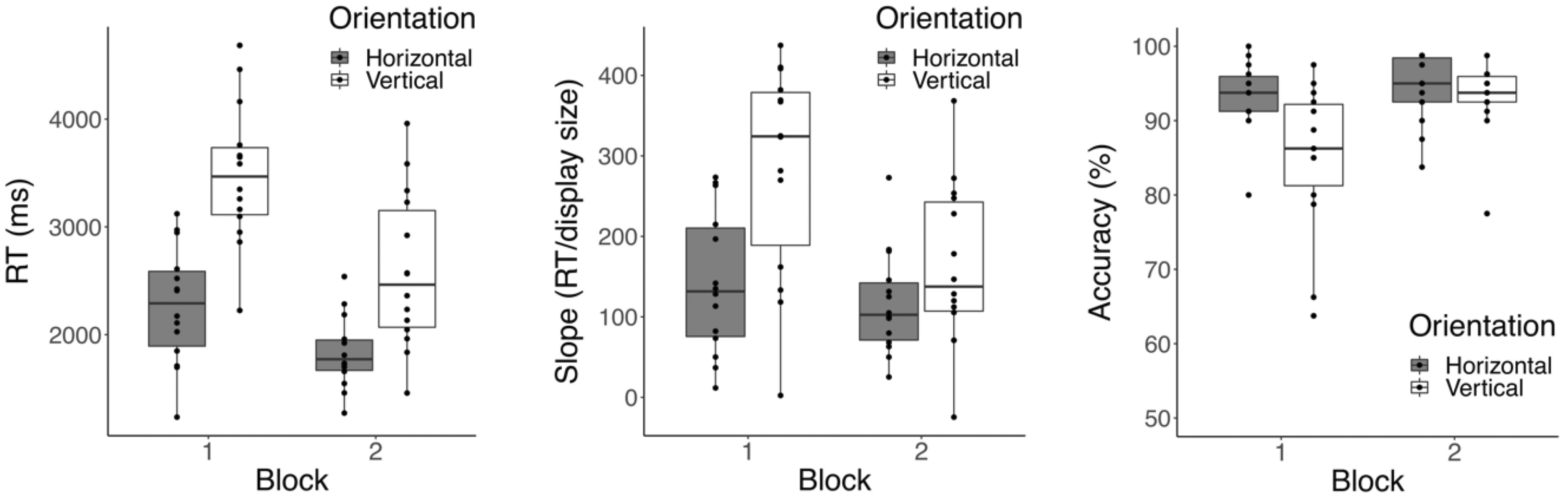
Performance as a function of orientation and block for: response time (left panel), search slope (middle panel), and accuracy (right panel). RT = response time.

Figure 3 shows how performance varied across the two blocks of interleaved trials for each of the dependent variables. There is clear evidence of task learning as both RT, *F*(1, 13) = 64.12, *p* < .001, $\eta_{p}^{2}$ = 0.83, and search slopes, *F*(1, 13) = 7.28, *p* = .02, $\eta_{p}^{2}$ = 0.36, decreased across block, while accuracy increases, *F*(1, 13) = 23.95, *p* < .001, $\eta_{p}^{2}$ = 0.65. Although Figure 3 suggests trends for the horizontal advantage to decrease in magnitude across block for both RT and slopes, neither of these Orientation × Block interactions reached significance (see OSF Supplementary Tables for details). Thus, the horizontal advantage for response time and search efficiency appears to be modulated, but not eliminated as a function of practice. For accuracy, the Orientation × Block interaction was significant, *F*(1, 13) = 7.10, *p* = .02, $\eta_{p}^{2}$ = 0.35, driven by a marked deficit in vertical responses during the first block of trials. The near ceiling levels of performance for horizontal responses in both blocks urges some caution in interpreting this pattern. As noted earlier, we did not design our task to explore accuracy as a primary dependent measure, rather it was included to check for speed-accuracy trade-offs. Nevertheless, the increased difficulty in initially detecting vertical targets, and the subsequent improvement with practice clearly has implications in terms of underlying mechanisms, a point we consider in more detail later. Full analysis tables for all tests have been uploaded to the OSF page associated with this article.

## Discussion

In this study, we tested whether searching for a horizontal target is faster and more efficient than searching for a vertical target. Using novel dynamic displays that masked low-level cues to orientation, we created a search task where orientation-specific search templates were required to guide attention in a top-down manner. As expected, search was overall slow and inefficient. More importantly, however, participants were almost a second faster in finding horizontal compared to vertical targets, a pattern seen in 13 of 14 data sets. They were also more efficient at searching for horizontal targets, reflected in shallower search slopes; and they were more accurate. To our knowledge, this is the first time a horizontal search advantage has been found in a direct comparison between horizontal and vertical targets, and both the magnitude of the horizontal advantage and its consistency across participants is intriguing.

How might such a search advantage arise? As participants almost certainly have to deploy attention serially across these displays via multiple fixations, it remains possible that our results reflect the operation of biased low-level orientation detectors. That is, although our dynamic displays may have prevented attention being draw automatically by such low-level visual mechanisms, they may nonetheless have exerted an influence on performance during each fixation. As mentioned in the Introduction, there is both neurophysiological (Coppola & White, 2004) and neuroimaging (Maloney & Clifford, 2015; Mannion et al., 2010) evidence to suggest biased early processing of horizontal orientations. Such biases could thus give rise to the present response time effects. One way to test this idea might be to interleave the current search displays with some form of orientation adaptation. For example, if we systematically fatigue horizontal orientation detectors by adapting them out at the start of a block, would this reduce or eliminate the current horizontal search advantage?

The fact that participants need to move their eyes in the current task also suggests additional measures that could be examined. Repeating this study while recording eye movements might yield several new insights. For example, both eye movement (Amor et al., 2016) and behavioural (Woods et al., 2013; Jóhannesson et al., 2016) measures indicate that participants typically scan complex search arrays in systematic ways, very often in left-to-right, top-to-bottom fashion, reminiscent of reading in Western cultures. Such scan paths might clearly favour horizontal targets in the current displays. There are individual differences in scanning behaviours, for example, some participants alternatively use top-to-bottom, left-to-right patterns, or have much less organised search. Quantifying the direction and strength of scan path biases in individuals and examining how these measures correlate with the size of the search advantage may indicate whether they play any role in the current task.

More generally, it is known that horizontal eye movements are not only faster and more precise but also more prevalent than vertical eye movements (Amor et al., 2016; Ke et al., 2013; Rottach et al., 1996). Such benefits for horizontal eye movements have been suggested to reflect adaptive responses to the environment in which horizontal directions are more relevant to us (Rottach et al., 1996). Exploring the general characteristics of eye movements with the current displays may thus prove useful. Alternatively, it may also be possible to adapt the current task so that it can be completed under fixation conditions. If a horizontal advantage still occurs, this would rule out the contribution of eye movements. Being able to parametrically vary target position relative to fixation would also make it possible to explore other orientation-related effects, such as the radial bias (Sasaki et al., 2006; Westheimer, 2003).

More generally, if a horizontal advantage persists under fixation conditions, this would implicate covert attentional mechanisms as the source of the current horizontal advantage. Attentional benefits for horizontal cue-target arrangements relative to vertical ones have been observed in various tasks and with different types of stimuli, ranging from Gabors to faces (Carrasco et al., 2004; Clevenger et al., 2019; Mackeben, 1999; Pilz et al., 2012; Tse et al., 2003; Zénon et al., 2009). These advantages do not seem to be restricted to the meridian but any horizontal cue-target arrangement within our visual field (Barnas & Greenberg, 2016; Clevenger et al., 2019; Pilz et al., 2012). So far, the precise mechanisms underlying such attentional benefits have not been identified, although some evidence suggests the involvement of cross-hemispheric communication (Greenberg et al., 2014; Reuter-Lorenz & Fendrich, 1990).

Returning to the current task, there are several ways in which the displays or procedure could be extended in order to shed further light on possible underlying mechanisms. Changing the absolute flicker frequency or the relative temporal frequency between target and surround might be one direction to explore. Our choice of a 10-Hz base frequency was arbitrary. Choosing absolute flicker frequencies based on the known properties of orientation detectors and/or examining whether the magnitude of the horizontal advantage remains constant as the temporal frequency modulates may prove useful. In the spatial domain, changing the aspect ratio of the dot grids themselves could be used to counteract or enhance the horizontal advantage. Finally, in the current task, there were indications that the horizontal advantage decreases with practice, particularly for accuracy (Figure 3). Reducing overall display duration to bring accuracy levels away from the ceiling may prove useful as a way to further explore learning patterns with this dependent variable. More generally, it may be interesting to examine whether extensive practice over 5 or 10 blocks, for example, is able to completely eliminate the horizontal advantage in all measures. If so, this would clearly implicate a learnt preference for horizontal orientations as the mechanism driving the current advantage, rather than more basic physiological mechanisms.

## Supplemental Material

sj-pdf-1-ipe-10.1177_20416695211004616 - Supplemental material for A Search Advantage for Horizontal Targets in Dynamic Displays Click here for additional data file. Supplemental material, sj-pdf-1-ipe-10.1177_20416695211004616 for A Search Advantage for Horizontal Targets in Dynamic Displays by Ian M. Thornton, Quoc C. Vuong and Karin S. Pilz in i-Perception
