# Supplementary material for: A Search Advantage for Horizontal Targets in Dynamic Displays
Source: Iperception. 2021 Apr 13;12(2):20416695211004616. doi: 10.1177/20416695211004616 (PMC8047869; doi:10.1177/20416695211004616)
Supplement: sj-pdf-1-ipe-10.1177_20416695211004616 - Supplemental material for A Search Advantage for Horizontal Targets in Dynamic Displays [file sj-pdf-1-ipe-10.1177_20416695211004616.pdf]

SUPPLEMENTARY TABLE 1

REACTION TIME

		SS	df	MS	F	Sig.	p-ETA
**Orientation**	Sphericity Assumed	54217416.11	1.00	54217416.11	28.51	0.00	0.69
Error(Orientation)	Sphericity Assumed	24718583.45	13.00	1901429.50			
**Block**	Sphericity Assumed	25109598.25	1.00	25109598.25	64.12	0.00	0.83
Error(Block)	Sphericity Assumed	5091060.06	13.00	391620.00			
**Display Size**	Sphericity Assumed	80687705.87	3.00	26895901.96	65.96	0.00	0.84
Error(SetSize)	Sphericity Assumed	15901791.07	39.00	407738.23			
**Orientation * Block**	Sphericity Assumed	3092835.00	1.00	3092835.00	4.29	0.06	0.25
Error(Orientation*Block)	Sphericity Assumed	9367439.06	13.00	720572.24			
**Orientation * Display Size**	Greenhouse-Geisser	6439410.58	1.75	3683743.29	3.08	0.07	0.19
Error(Orientation*Display Size)	Greenhouse-Geisser	27145377.10	22.72	1194526.44			
**Block * Display Size**	Greenhouse-Geisser	5826262.16	1.82	3206322.37	4.49	0.03	0.26
Error(Block*Display Size)	Greenhouse-Geisser	16859621.78	23.62	713709.76			
**Orientation * Block * Display Siz**	Sphericity Assumed	1740160.76	3.00	580053.59	1.23	0.31	0.09
Error(Orientation*Block*Display	Sphericity Assumed	18397576.42	39.00	471732.73			

ERRORS

		SS	df	MS	F	Sig.	p-ETA
**Orientation**	Sphericity Assumed	0.12	1	0.12	5.96	0.03	0.31
Error(Orientation)	Sphericity Assumed	0.26	13	0.02			
**Block**	Sphericity Assumed	0.11	1	0.11	23.95	0.00	0.65
Error(Block)	Sphericity Assumed	0.06	13	0.00			
**Display Size**	Sphericity Assumed	0.19	3	0.06	18.10	0.00	0.58
Error(SetSize)	Sphericity Assumed	0.14	39	0.00			
**Orientation * Block**	Sphericity Assumed	0.07	1	0.07	7.10	0.02	0.35
Error(Orientation*Block)	Sphericity Assumed	0.12	13	0.01			
**Orientation * Display Size**	Sphericity Assumed	0.05	3	0.02	4.94	0.01	0.28

Error(Orientation*Display Size)	Sphericity Assumed	0.13	39	0.00			
**Block * Display Size**	Sphericity Assumed	0.01	3	0.00	1.60	0.20	0.11
Error(Block*Display Size)	Sphericity Assumed	0.11	39	0.00			
**Orientation * Block * Display Siz**	Greenhouse-Geisser	0.03	1.74	0.02	2.57	0.10	0.16
Error(Orientation*Block*Display	Greenhouse-Geisser	0.17	22.66	0.01			

SLOPE

		SS	df	MS	F	Sig.	p-ETA
**Orientation**	Sphericity Assumed	1129098.80	1	1129098.80	13.91	0.00	0.52
Error(Orientation)	Sphericity Assumed	1055326.62	13	81178.97			
**Block**	Sphericity Assumed	688356.35	1	688356.35	7.28	0.02	0.36
Error(Block)	Sphericity Assumed	1229900.00	13	94607.69			
**Orientation* Block**	Sphericity Assumed	295031.89	1	295031.89	2.48	0.14	0.16
Error(Orientation* Block)	Sphericity Assumed	1549078.05	13	119159.85			
